# Real-World User Demographics of Three Web-Based Digital Mental Health Interventions Provided by the US Department of Veterans Affairs: Observational Study Using Web Analytics Data

**DOI:** 10.2196/48365

**Published:** 2023-10-18

**Authors:** Arthur T Ryan, Kelly A Stearns-Yoder, Lisa A Brenner

**Affiliations:** 1 Rocky Mountain Mental Illness Research, Education and Clinical Center for Suicide Prevention Department of Veterans Affairs Rocky Mountain Regional Veterans Affairs Medical Center Aurora, CO United States; 2 Department of Psychiatry University of Colorado Anschutz Medical Campus Aurora, CO United States; 3 Department of Physical Medicine and Rehabilitation University of Colorado Anschutz Medical Campus Aurora, CO United States; 4 Department of Neurology University of Colorado Anschutz Medical Campus Aurora, CO United States

**Keywords:** digital intervention, unguided, web-based, internet-delivered, mental health, veterans, Google Analytics, insomnia, anger, depression, mobile phone

## Abstract

**Background:**

Unguided digital mental health interventions (UDMHIs) have the potential to provide low-cost and effective mental health care at scale. Controlled trials have demonstrated the efficacy of UDMHIs to address mental health symptoms and conditions. However, few previous publications have described the demographics of real-world users of UDMHIs that are freely available to the public. The US Department of Veterans Affairs has created and hosts several UDMHIs on its Veteran Training Portal website. These web-based, free-to-use, and publicly available UDMHIs include *Path to Better Sleep*, *Anger and Irritability Management Skills*, and *Moving Forward*, which focus on insomnia, problematic anger, and depression symptoms, respectively.

**Objective:**

This study aimed to examine the user demographics of these 3 UDMHIs in the year 2021. In addition, it aimed to compare the age and gender distribution of the users of those 3 UDMHIs with one another and with the age and gender distribution of the total US veteran population.

**Methods:**

Google Analytics was used to collect user data for each of the 3 UDMHIs. The age and gender distribution of the users of each UDMHI was compared with that of the other UDMHIs as well as with that of the overall US veteran population using chi-square tests. Information on the total number of users, the country they were in, and the devices they used to access the UDMHIs was also collected and reported.

**Results:**

In 2021, the 3 UDMHIs together recorded 29,306 unique users. The estimated age range and gender were available for 24.12% (7068/29,306) of those users. Each UDMHI’s age and gender distribution significantly differed from that of the other UDMHIs and from that of the overall US veteran population (*P*<.001 on all chi-square tests). Women and younger age groups were overrepresented among UDMHI users compared with the overall US veteran population. The majority of devices used to access the UDMHIs were desktop or laptop devices, although a substantial proportion of devices used were mobile devices (10,199/29,752, 34.28%). Most users (27,789/29,748, 93.41%) were located in the United States, with users from Canada, the United Kingdom, and Australia accounting for another 2.61% (775/29,748) of total users.

**Conclusions:**

Our use of Google Analytics data provided useful information about the users of 3 free and publicly available UDMHIs provided by the US Department of Veterans Affairs. Although our findings should be considered in light of the limitations of autonomously collected web analytics data, they still offer useful information for health care policy makers, administrators, and UDMHI developers.

## Introduction

### Unguided Digital Mental Health Interventions

Digital mental health interventions are software programs designed to improve mental health symptoms and conditions or otherwise improve mental health [1]. These interventions can be delivered via website, mobile app, or desktop application. These interventions can be guided, meaning that their use is facilitated by interacting with another person (usually a health care provider), or unguided, meaning that their use is not facilitated by another person. Unguided digital mental health interventions (UDMHIs) have been referred to by several names in the literature, including web-based interventions [2] and digital self-help interventions [3]. Web-based UDMHIs have the potential to provide effective treatment to the hundreds of thousands of individuals in the United States and worldwide who do not receive evidence-based psychotherapies for their mental health conditions because of a variety of treatment barriers, including cost and the limited availability of health care providers trained in the use of those psychotherapies [4-6]. Meta-analyses and systematic reviews of controlled trials of UDMHIs have found that they are effective in treating a variety of highly prevalent and often disabling mental health conditions, including depression, anxiety, and insomnia disorders [7,8]. Consistent with the evidence for their effectiveness, UDMHIs have been recommended as standard treatments in some national guidelines for mental health conditions [9].

### Existing Studies of the Real-World Use of UDMHIs

As previously noted, meta-analyses of controlled clinical trials have supported the efficacy of UDMHIs [7,8]. However, to confirm the feasibility, scalability, and effectiveness of UDMHIs in real-world clinical use, it is important to supplement controlled clinical trials with studies of the real-world use of UDMHIs. For example, it is important to know how many individuals actually use publicly available UDMHIs and whether those users are similar to the individuals who participate in controlled trials of UDMHIs [10]. Few controlled UDMHI trials have examined the representativeness of their participants compared with the wider populations of individuals that the UDMHIs are intended to serve [10-12]. Indeed, those few existing studies suggest that real-world UDMHI users and trial participants may differ in important ways [10,13-15]. Differences between a UDMHI’s real-world users and trial participants might potentially help explain instances in which a UDMHI was found to be efficacious in a controlled clinical trial but was subsequently found to be less effective when deployed in a less controlled trial or in a real-world clinical setting [3,16].

Although the participants in dozens of published trials of UDMHIs for depression and anxiety have been described [17,18], relatively few studies have been published describing the individuals who use UDMHIs when they are made publicly available. A 2018 systematic review of studies on user uptake and engagement with publicly available UDMHIs for depression and anxiety identified 10 such studies in adults [3]; only 5 of those 10 studies reported the gender and mean or median age of the UDMHIs’ users [19-23], with 2 more reporting either age or gender [24,25].

In this paper, we attempt to add to the literature describing real-world users of free, publicly available, web-based UDMHIs by describing the users of 3 such UDMHIs hosted by the US Department of Veterans Affairs (VA): *Path to Better Sleep* (PTBS), *Anger and Irritability Management Skills* (AIMS), and *Moving Forward* (MF). These 3 UDMHIs are available on the Veteran Training Portal (VTP), a subdomain of the official VA website. We wish to be clear that this study focuses on the demographics of users of these UDMHIs and not on their use behaviors when accessing the UDMHIs (eg, the average length of time users spent viewing the website). As this paper is one of the first to describe these VA UDMHIs, we will also provide a description of the content and development of these UDMHIs.

### Descriptions of the VTP and 3 UDMHIs

#### Veteran Training Portal

The VTP hosts UDMHIs that are made freely available to veterans and members of the general public. Over time, new UDMHIs have been added to the VTP, and the existing ones have been updated. The 3 UDMHIs reported on in this paper share several key characteristics, which are described in Textbox 1. In particular, they all deliver adaptations of existing evidence-based mental health treatments (eg, cognitive behavioral therapy for insomnia [CBT-I]). The VTP also hosts a few other web-based programs that focus on psychoeducation and training (eg, *Parenting for Veterans*, a collection of videos, written material, and worksheets to improve parenting skills [26]). We will focus on the 3 previously mentioned UDMHIs as they were the 3 fully featured and comprehensive UDMHIs that were available on the VTP in 2021.

**General information about the development and characteristics of unguided digital mental health interventions (UDMHIs) hosted on the Veteran Training Portal (VTP).**

- Veterans Health Administration (VHA) leadership identifies a clinical need (eg, untreated insomnia symptoms in veterans) and allocates resources for the production of a web-based UDMHI that addresses that need.
- VHA staff (sometimes in collaboration with members of other federal agencies such as the Department of Defense) oversee the development of the UDMHI.
- Subject matter experts within and outside the US Department of Veterans Affairs (VA) are recruited to select existing VA materials to adapt as well as to generate new content to be included in the UDMHI.
- Web design, illustration, and programming are carried out by a software developer contracted by the VHA.
- The UDMHIs hosted on the VTP include a variety of content delivered via an internet browser–based user interface, such as the following:
  Videos of clinicians introducing the intervention and key concepts
  Videos of veterans describing their experiences with the intervention (usually with the traditional in-person implementation of the intervention that the UDMHI is based on) or giving advice to the user on how to handle more challenging aspects of the UDMHI (eg, how to complete practice assignments effectively)
  Animations explaining intervention content
  Questionnaires and assessment measures that provide feedback to the user
  Graphical illustrations
  Text
  Interactive activities that provide the opportunity to practice applying material learned in the course (eg, matching definitions to key terms, selecting advice to provide to characters in vignettes)
  Forms that can be filled out, including intervention worksheets
- Data generated by veterans are stored locally as a cookie and can be printed out.
- None of the hosted interventions require a user log-in. None of them store individual user treatment data on VA servers. Thus, no personal health information is stored on remote servers and no infrastructure is needed to store individual users’ health data.

#### Path to Better Sleep

PTBS is a UDMHI adaptation of CBT-I [27]. In 2023, PTBS went through a rebranding process. The name of the CBT-I UDMHI was changed to “SleepEZ,” whereas “Path to Better Sleep” now instead refers to a package of several web-based sleep-related programs. As the CBT-I UDMHI was titled *Path to Better Sleep* in 2021, we will refer to it as such for the remainder of this paper. PTBS is based on a self-guided CBT-I treatment workbook titled *Improve your Sleep: A Self-Guided Approach for Veterans with Insomnia* [28]. This workbook was, in turn, adapted from an existing manualized implementation of CBT-I developed for the VA [29].

Before the creation of PTBS, the VA carried out a large manualized CBT-I treatment dissemination program throughout its facilities; this dissemination program included the creation of standardized training materials and the deployment of dedicated training staff [30]. This dissemination program trained >1000 VA providers in CBT-I, and veterans treated by these providers have experienced substantial improvements in insomnia and depression symptoms [31]. However, despite this large effort, the availability of CBT-I providers continues to limit the number of veterans who can receive CBT-I, particularly among the many veterans who receive care outside of the VA [32,33]. This limitation in the availability of CBT-I was a key motivation for the development of PTBS. All CBT-I implementations, including PTBS, include several key therapeutic components [34], which are described in Textbox 2.

**Summary of key concepts and skills taught in Path to Better Sleep and other cognitive behavioral therapy (CBT) for insomnia–based interventions.**

- Psychoeducation regarding sleep and its physiology (eg, sleep drive and circadian processes) and the CBT model of insomnia
- Stimulus control, which is a set of classical conditioning-informed practices that associate one’s bed with sleep (eg, reserving the bed solely for sleep and sexual intimacy, only laying down in bed when sleepy, and leaving the bed after 20 min if the individual fails to fall asleep)
- Sleep restriction, which decreases the fragmentation and increases the quality of sleep by creating a “sleep prescription” that restricts time spent in bed to the actual amount of time spent sleeping in the previous week
- Sleep hygiene (eg, avoidance of alcohol and caffeine before bed)
- Relaxation techniques such as mindful breathing
- Cognitive therapy exercises to address cognitions contributing to insomnia; examples of such exercises are the following:
  Scheduling “worry time” before bed
  Challenging unrealistic or unhelpful sleep-related beliefs (eg, “In order to get to sleep, I must try really hard.”)

In several meta-analyses, digital intervention adaptations of CBT-I were found to have similar efficacy to traditionally delivered CBT-I [7]. A paper has been published describing feedback from evaluation panels on drafts of PTBS’s content during its development [35]. At the time of writing, two 2-armed randomized controlled trials are underway: one comparing teleprovider-supported use of the self-help workbook that PTBS is based on with a teleprovider-supported health education control [36,37] and the other evaluating the efficacy of guided PTBS among those with moderate to severe traumatic brain injury and insomnia [38]. The authors are unaware of any other studies that have examined the acceptability, feasibility, or efficacy of PTBS itself.

#### Anger and Irritability Management Skills

AIMS is a UDMHI adaptation of cognitive behavioral therapy (CBT) for anger management [39]. Specifically, AIMS’s content was adapted from *Anger Management for Substance Abuse and Mental Health Clients*, a 12-week CBT group therapy protocol for the treatment of problematic anger that is freely available from the US Substance Abuse and Mental Health Services Administration [40]. Several changes were made to the content of the original CBT treatment protocol when it was adapted into the AIMS UDMHI. In total, 4 of the 12 sessions in the original treatment protocol focused on reviewing and practicing previously introduced content [40]. After abridging these sessions and rearranging the order in which some concepts are introduced, the AIMS course now consists of 8 modules rather than the original 12 sessions. A summary of the key concepts and skills of AIMS (as well as the original therapy protocol) is presented in Textbox 3.

**Summary of key concepts and skills taught in Anger and Irritability Management Skills.**

- Psychoeducation about the cognitive behavioral therapy conceptualization of anger and aggressive behavior
- Relaxation techniques to reduce the emotional and physiological components of anger (eg, a deep breathing exercise)
- Cognitive techniques targeting cognitive processes related to anger, including hostile appraisals and attributions, irrational beliefs, and inflammatory thinking (eg, thought stopping to interrupt ruminative angry thoughts)
- Communication skills that help the individual engage in effective assertiveness and conflict resolution behaviors (eg, step-by-step instructions for raising a disputed issue with another person effectively along with structured practice of this technique)

The *Anger Management for Substance Abuse and Mental Health Clients* treatment protocol has been found to be effective for the reduction of problematic anger in several individual treatment trials [41], including trials with veterans receiving care in the VA system [42]. Meta-analyses have found that CBT therapies overall are effective for the treatment of problematic anger [43,44]. The authors are unaware of any studies that have examined the acceptability, feasibility, or efficacy of the AIMS UDMHI itself. The authors are also unaware of published studies on UDMHI adaptations of CBT for problematic anger.

#### Moving Forward

The MF UDMHI is an adaptation of an existing group therapy protocol with the same name. The original MF protocol is based on problem-solving therapy [45], which is a psychosocial intervention generally considered to be consistent with the CBT model [46]. The VA commissioned the creation of the original MF protocol, and that protocol was subsequently disseminated to VA providers through a standardized training program [45]. The original MF protocol is delivered in 4 sessions over 4 weeks, with a standard group size of 5 or 6 veterans. The goals of the original MF protocol are to reduce depression symptoms, improve the attainment of life goals, and buffer individuals against the effects of stress. The UDMHI adaptation of the original MF protocol was built by the VA in partnership with the Department of Defense [47]. It consists of 5 modules: the first 4 modules cover the content of the 4 sessions of traditional MF, whereas the fifth module is a short wrap-up session summarizing the course and how the individual can continue to practice the skills they have learned. The key concepts and skills taught in MF are summarized in Textbox 4.

**Summary of key concepts and skills taught in Moving Forward.**

- Psychoeducation about the problem-solving therapy conceptualization of negative emotions and related functional impairments, including the following:
  The existence of individual problem-solving styles
  The fact that stress and complex problems tend to cause “cognitive overload,” where an individual is unable to manage the information needed to solve complex problems or make effective decisions
  The fact that negative emotions both contribute to and are caused by cognitive overload
- Skills for managing cognitive overload, including the following:
  Externalization of the relevant information using writing, diagrams, charts, and lists
  Visualization (ie, deliberately simulating aspects of the problem in the mind’s eye to clarify them) and mental rehearsal of carrying out a solution
  Breaking up a complex problem into several smaller, simpler ones and translating abstract or vague aspects of the problem into concrete terms
- Skills for managing negative arousal, including the following:
  “Stop, Slow Down, Think, and Act,” a series of steps practiced when the individual notices negative arousal
- A formal process for problem-solving, which includes defining the problem, generating alternative solutions, deciding on a solution to attempt, attempting the solution, and assessing its results

Several studies of MF have been published. A program evaluation of the traditional MF group therapy delivered to veterans receiving care in the VA found significant pretreatment to posttreatment improvement on self-reported measures of depression symptoms, general psychopathology, resilience, and problem-solving skills, along with positive results on measures of program satisfaction and attrition [45]. A small trial of a version of traditional MF adapted for use in a primary care setting also reported positive results for symptom improvement and acceptability [48]. Another small pilot trial tested an individual therapy adaptation of the traditional MF protocol where the first session was held in person and the remainder were held over the telephone—this trial showed moderate effect size estimates for pretreatment to posttreatment change in depression symptoms [49]. A recently published pilot study described a trial of the MF UDMHI adaptation and included both peer-supported and unguided treatment arms [47]. In this pilot study, veterans in both the peer-supported and unguided conditions showed significant decreases in pretreatment to posttreatment depression symptoms.

## Methods

### Data Collection

The data reported in this paper were collected as part of normal website operations from users who visited the UDMHI web pages between January 1, 2021, and December 31, 2021. The same data variables were collected for each of the 3 UDMHIs. Data were collected using Google Analytics [50]. Google Analytics is a web analytics tool that collects, analyzes, and reports data on the users of a website—these data are usually used to understand and optimize visitor engagement with that website. As of March 2022, Google Analytics is used by more than half of all existing websites, making it the most widely used web analytics platform [51]. Google Analytics data have been used in studies of mental health–related websites, including a youth-oriented mental health web portal [52], a men’s depression resource website [53], and a smoking cessation program [54]. Google Analytics has also been used to study other websites attempting to change health-related behaviors, including sexual health behaviors [55] and antibiotic use [56]. Google Analytics works via the inclusion of a block of JavaScript code in a web page [57]. When a user visits the web page, the JavaScript code is executed and collects data on the request sent by the user’s browser to the web page’s server. These data are then sent to a Google Analytics server for processing and analysis. The collected data include the user’s http request and information from first-party (ie, Google created and owned) cookies. The user’s http request for the original web page includes details about the web browser and the device making the request (eg, whether it is a standard desktop or laptop computer or a mobile device such as a smartphone), the referrer (eg, whether the user reached the website by typing in the web address manually or clicking a hyperlink), and the country in which the individual was located when they accessed the course (deduced from the IP address of the user).

The Google Analytics code embedded in the 3 VA UDMHIs was also configured to attempt to deduce the demographics of visitors to those websites. Google Analytics can deduce 2 demographic variables: gender (man and woman categories only) and age bracket (18-24, 25-34, 35-44, 45-54, 55-64, and ≥65 y). To make this deduction, Google Analytics examines a cookie or advertising ID (ie, the DoubleClick advertising network cookie, the Android advertising ID, or the iOS identifier for advertisers) [58]. The cookie or ID contains information relevant to the user’s demographics and interests—this can include information that the user has either directly entered during their website browsing (eg, by explicitly reporting their gender to a website) or that has been estimated based on patterns in their web use (eg, making purchases or visiting websites more frequently associated with a particular gender or age group). If the user is logged into their Google account while browsing the web (eg, is logged into Gmail or using the Chrome browser with their user profile logged in), this information also includes data gathered from their use of Google services [59,60]. If Google Analytics algorithmically determines that it does not have sufficient data to validly estimate a user’s gender or age range, it will not provide an estimate for that user’s demographic variables. A user’s estimated demographic data are normally used to help target advertisements to that user and provide feedback to the website owner on the demographic makeup of users of their website. A study comparing users’ gender and age estimated by Google Analytics with self-reported values found that, among the subset of individuals for whom Google Analytics made a prediction, those estimates were fairly accurate for both gender (90% accuracy) and age bracket (79% accuracy) [60]. Tables S1 and S2 in Multimedia Appendix 1 provide details regarding these accuracy calculations.

For users without an existing DoubleClick cookie or device ID, a cookie (named “_ga”) is saved on the user’s computer containing a unique client ID code. This cookie is used to differentiate between returning users and those accessing the website for the first time. As with all browser cookies, users may block the “_ga” cookie’s creation or delete it after it is created. Thus, the unique user count computed using browser cookies is necessarily imprecise as various forms of user behavior (eg, multiple users using the same computer or users deleting cookies in between sessions) may alter the estimated user count [61].

### Ethical Considerations

A protocol for the preparation of this manuscript was submitted to the VA offices responsible for reviewing proposed quality improvement and program evaluation projects. It was concluded that the preparation of this manuscript did not fall within the category of research as it dealt only with nonsensitive, aggregate, and unidentified data collected as part of the normal operation of the websites in question. Thus, appropriate approvals were granted for the preparation and submission of this manuscript for publication. Furthermore, no institutional review board review was required given that the preparation of this manuscript was not found to constitute human participant research.

### Statistical Analysis

Counts, percentages, and means were computed to describe UDMHI users. Chi-square tests were conducted to determine whether the age and gender distribution of users of each UDMHI differed from that of users of the other UDMHIs as well as from the age and gender distribution of the total US veteran population. The estimated age and gender distribution of the total US veteran population in 2021 was obtained from the Veteran Population Projection Model 2018 data set [62], which is freely available from the National Center for Veterans Analysis and Statistics [63]. We chose to compare the demographics of the UDMHIs’ users to those of the entire veteran population (as opposed to the demographics of veterans receiving Veterans Health Administration care and diagnosed with the mental health conditions targeted by the individual UDMHIs) because (1) the UDMHIs were intended to be used by all veterans regardless of whether they receive care within the Veterans Health Administration and (2) the UDMHIs are also intended for use by individuals with symptoms of the disorders in question but who do not meet the full criteria for the relevant disorder.

## Results

The data reported in this paper were collected from users who visited the UDMHI web pages between January 1, 2021, and December 31, 2021. Table 1 summarizes the number of users of each UDMHI. Table 1 also summarizes the number of each kind of device that was used to connect to the course (a single user could use multiple devices to connect to the course across sessions, so the total number of unique visitors [N=29,306] is slightly smaller than the total number of devices [N=29,752] used to connect to the course). Although the number of users of PTBS (n=6613) and MF (n=6693) was similar, the number of users of AIMS (n=16,000) was more than twice that of the other 2 UDMHIs in 2021. Across UDMHIs, the devices most often used to access the websites were desktop or laptop computers (18,528/29,752, 62.27% of all devices) followed by mobile devices (10,199/29,752, 34.28% of all devices), with only a small minority of devices being tablets (1025/29,752, 3.45% of all devices).

**Table 1:** Number of users and demographic information for the US Department of Veterans Affairs unguided digital mental health interventions (UDMHIs) (N=29,306).

			PTBS^a^ (n=6613), n (%)		AIMS^b^ (n=16,000), n (%)		MF^c^ (n=6693), n (%)		Total, n (%)
Users with demographics estimated			1552 (23.47)		4010 (25.06)		1506 (22.5)		7068 (24.12)
Woman users^d^			737 (47.49)		1658 (41.35)		774 (51.39)		3169 (44.84)
**Devices used to connect to the UDMHIs by type (N=29,752)^e^**									
	Desktop or laptop computer	4526 (68.17)		9381 (58.2)		4621 (66.07)		18,528 (62.27)	
	Mobile phone	1869 (28.15)		6196 (38.44)		2134 (30.51)		10,199 (34.28)	
	Tablet	244 (3.68)		542 (3.36)		239 (3.42)		1025 (3.45)	

^a^PTBS: Path to Better Sleep.

^b^AIMS: Anger and Irritability Management Skills.

^c^MF: Moving Forward.

^d^Percentages in this row are percentages of the subset of users who had demographics available.

^e^Users could connect to the UDMHIs with more than 1 device over multiple sessions. Thus the total number of devices used to access the course (N=29,752) is slightly larger than the number of total users of the course (N=29,306).

**Figure 1:**
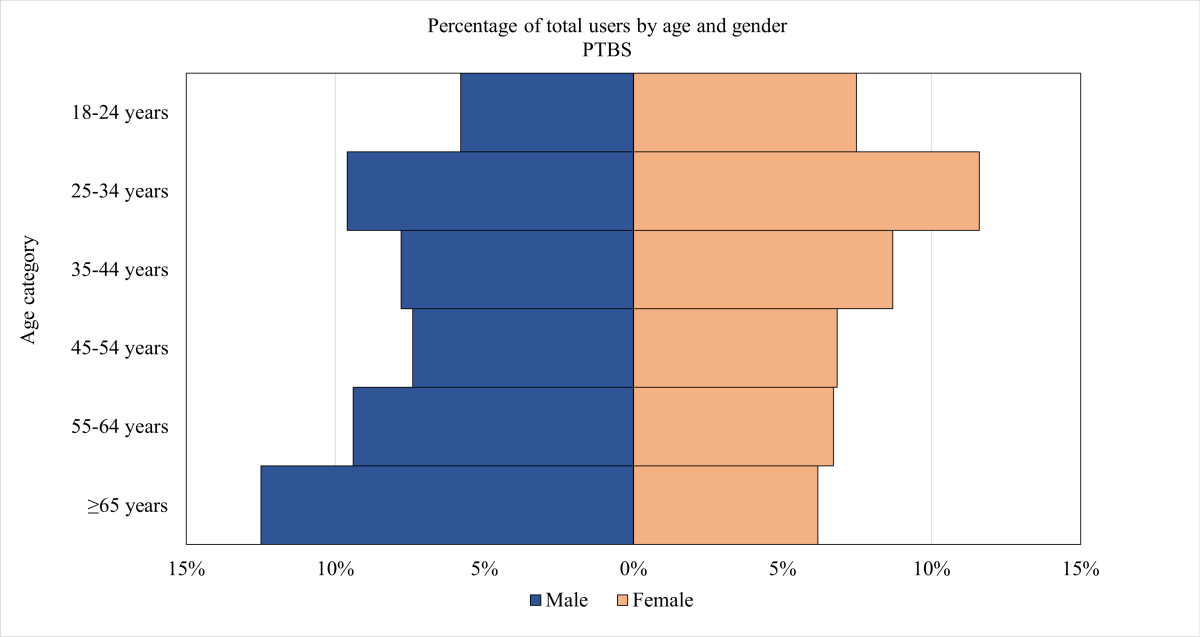
Path to Better Sleep (PTBS) population pyramid for Google Analytics–estimated demographics of users visiting the PTBS website in 2021. Percentages shown are of users with estimated gender and age data available.

**Figure 2:**
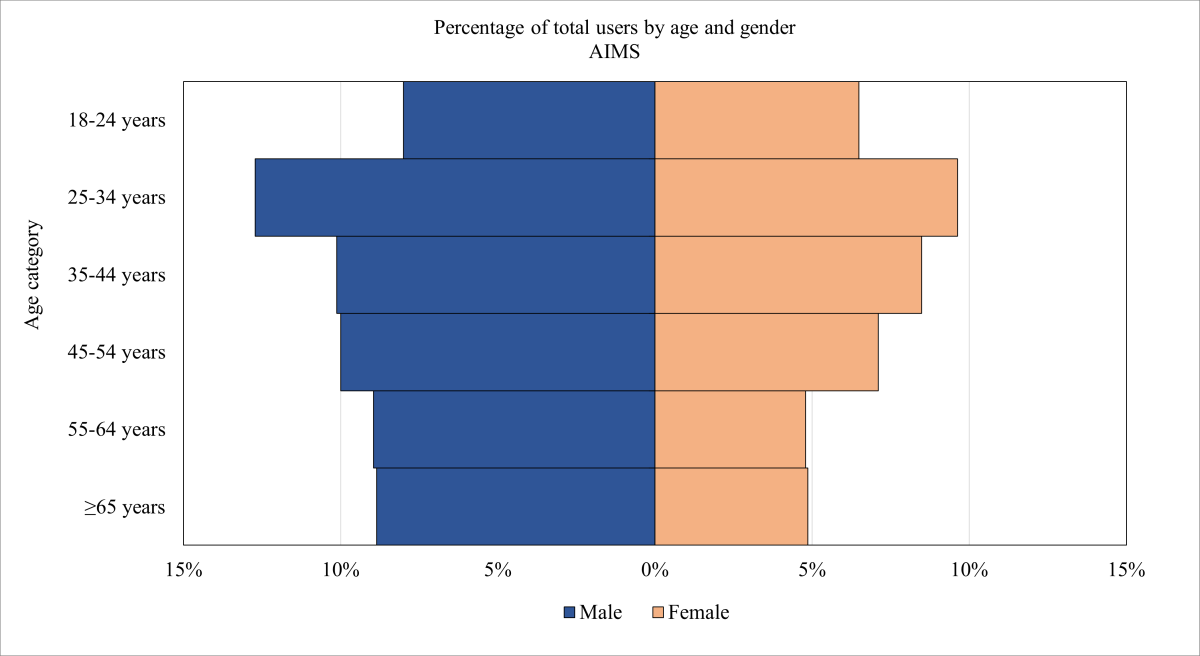
Anger and Irritability Management Skills (AIMS) population pyramid for Google Analytics–estimated demographics of users visiting the AIMS website in 2021. Percentages shown are of users with estimated gender and age data available.

**Figure 3:**
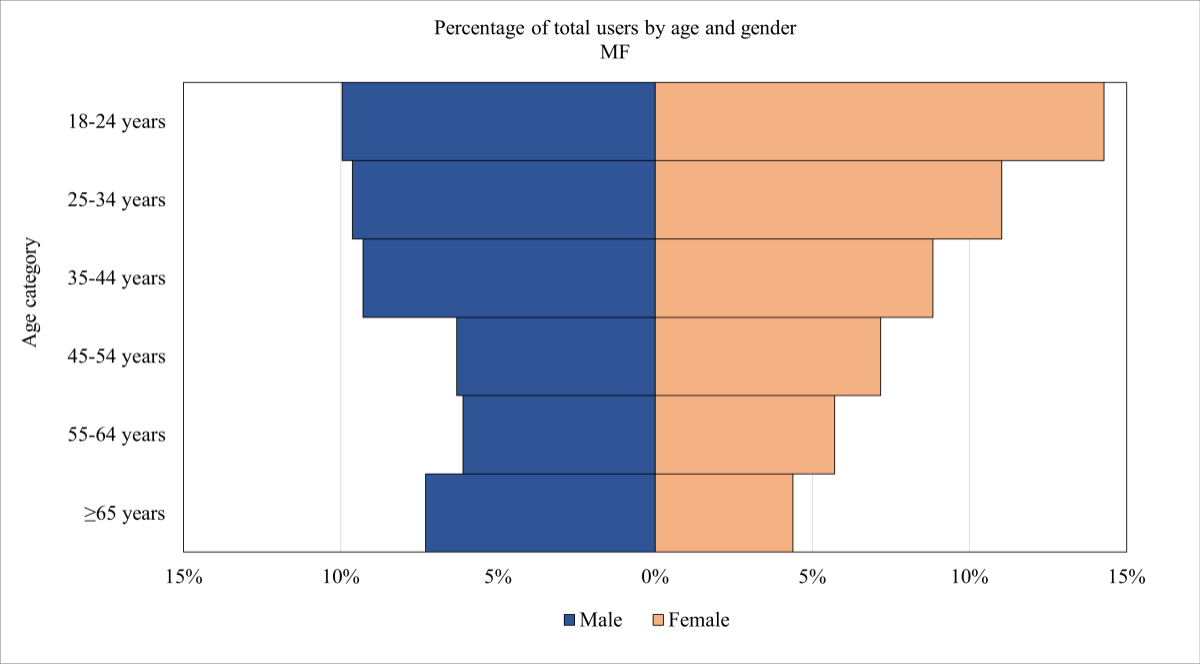
Moving Forward (MF) population pyramid for Google Analytics–estimated demographics of users visiting the MF website in 2021. Percentages shown are of users with estimated gender and age data available.

**Figure 4:**
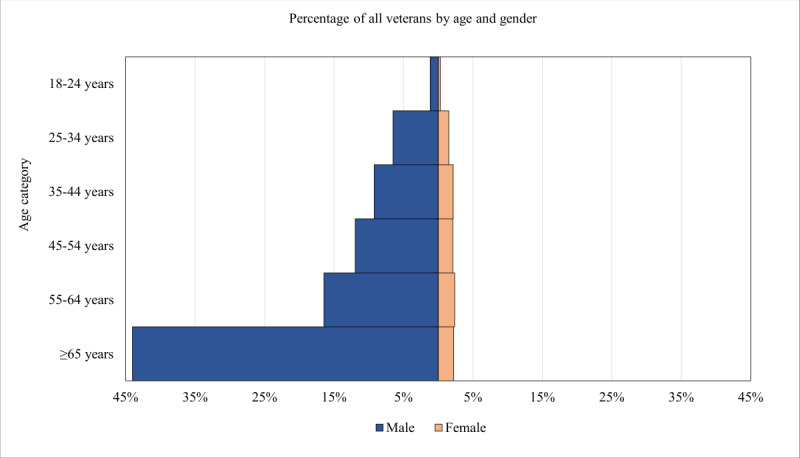
Population pyramid of the total US veteran population in 2021 using data from the Veteran Population Projection Model 2018 data set.

As shown in Table 1, the percentage of users for whom Google Analytics provided estimates of age and gender was similar across PTBS (1552/6613, 23.47%), AIMS (4010/16,000, 25.06%), and MF (1506/6693, 22.5%). Figures 1-3 are population pyramids illustrating the age and gender distribution of the users of each UDMHI (among users with an age and gender estimate available). Figure 4 is a population pyramid illustrating the age and gender distribution of the entire US veteran population in 2021. Table S3 in Multimedia Appendix 1 provides the number of individuals in each of the gender and age categories for the 3 UDMHIs and for the total US veteran population. Table 2 presents the results of chi-square tests comparing the age and gender distribution of the users of the UDMHIs with one another as well as with that of the entire US veteran population. As shown in Table 2, all these chi-square tests were significant (*P*<.001 in all cases), indicating that the age and gender category distribution of each UDMHI significantly differed from that of the other UDMHIs and from that of the entire US veteran population.

**Table 2:** Chi-square tests comparing the age and gender distributions of US Department of Veterans Affairs unguided digital mental health interventions’ 2021 users and of the entire 2021 US veteran population^a^.

Groups compared	Chi-square (*df*)	*P* value
PTBS^b^ vs total US veteran population	5164 (11)	<.001
PTBS vs AIMS^c^	63.9 (11)	<.001
PTBS vs MF^d^	90.6 (11)	<.001
AIMS vs total US veteran population	11,470 (11)	<.001
AIMS vs MF	127 (11)	<.001
MF vs total US veteran population	12,958 (11)	<.001

^a^Demographic values for the total US veteran population in 2021 were drawn from the publicly available Veteran Population Projection Model 2018 data set.

^b^PTBS: Path to Better Sleep.

^c^AIMS: Anger and Irritability Management Skills.

^d^MF: Moving Forward.

Visually comparing the 3 UDMHIs’ user population pyramids revealed several differences. Men made up a larger percentage of AIMS users (2352/4010, 58.65%) than of PTBS users (815/1552, 52.51%) and MF users (732/1506, 48.61%). The distribution among age categories also varied across the UDMHIs, with users aged 18 to 44 years (ie, the 3 younger age categories) accounting for 50.97% (791/1552) of PTBS users, 55.44% (2223/4010) of AIMS users, and 63.01% (949/1506) of MF users. PTBS’s largest demographic category was men aged ≥65 years (194/1552, 12.5%), AIMS’s largest demographic category was men aged 25 to 35 years (510/4010, 12.72%), and MF’s largest demographic category was women aged 18 to 24 years (215/1506, 14.28%).

However, all the differences among the 3 UDMHIs’ age and gender distributions were dwarfed by their differences from the total US veteran population age and gender distribution. Men aged ≥65 years made up 44.04% (8,439,505/19,162,515) of the total US veteran population compared with 12.5% (194/1552) of PTBS users, 8.85% (355/4010) of AIMS users, and 7.3% (110/1506) of MF users. The combined users of all 3 UDMHIs were 44.84% (3169/7068) women compared with only 10.67% (2,045,384/19,162,515) of the total US veteran population. Users aged 18 to 44 years made up 56.07% (3963/7068) of the users of the 3 UDMHIs but only 20.83% (3,992,206/19,162,515) of the total US veteran population.

Table 3 shows the countries in which the users were when they connected to the 3 UDMHIs. Across all 3 UDMHIs, most users (27,789/29,681, 93.63%) were located in the United States, with an additional 2.61% (775/29,681) of users located in the 3 next most common countries (Canada, the United Kingdom, and Australia) combined. Note that the total number of users for country location calculations was slightly higher (29,681) than the total number of unique users (29,306) because a unique user who logged in from multiple countries across separate sessions would be counted twice in the country log-in calculations.

**Table 3:** Location of users of US Department of Veterans Affairs unguided digital mental health interventions in 2021 (N=29,306)^a^.

Rank	Country	Users, n		PTBS^b^, n		AIMS^c^, n		MF^d^, n	
1	United States	27,789		6194		15,024		6571	
2	Canada	431		128		258		45	
3	United Kingdom	212		40		150		22	
4	Australia	132		9		100		23	
5	Unknown	100		30		49		21	
6	India	94		3		67		24	
7	South Africa	92		5		83		4	
8	Puerto Rico	72		9		35		28	
9	Philippines	63		4		24		35	
10	Japan	50		6		33		11	
All other countries			646		155		302		189

^a^The total number of visitors logging in from all countries (including visitors whose country could not be deduced from their IP address) is 29,681, which is larger than the total number of unique visitors we have reported earlier in this manuscript (n=29,306). The reason this number is larger is because a small subset of unique visitors logged in from IP addresses associated with 2 (or more) different countries. Such visitors are counted twice for the purpose of this table. For example, if a visitor logged in from an IP address associated with the US and then later from 1 associated with Canada, they would add 1 to the count of both US visitors and Canadian visitors.

^b^PTBS: Path to Better Sleep.

^c^AIMS: Anger and Irritability Management Skills.

^d^MF: Moving Forward.

## Discussion

### Principal Findings

#### Overview

In this manuscript, we describe the individuals who visited 3 web-based UDMHIs between January 1, 2021, and December 31, 2021. These 3 UDMHIs were hosted by the VA and were freely available to anyone with access to the internet. Nearly 30,000 unique users were estimated to have visited these 3 UDMHI websites. Google Analytics provided estimated age and gender data for 24.12% (7068/29,306) of those users. Nearly half (3169/7068, 44.84%) of the UDMHI users with demographic information available were women, all age categories (18-24, 25-34, 35-44, 45-54, 55-64, and ≥65 y) were well represented, most individuals (18,528/29,752, 62.27%) used desktop or laptop computers to access the UDMHIs, and nearly all users (27,789/29,681, 93.41%) were in the United States. Each UDMHI’s user age and gender distribution differed significantly from that of the others. Moreover, each UDMHI’s user age and gender distribution differed even more markedly from that of the total US veteran population. In this section, we will review our findings in the context of existing research and discuss how our study and findings might inform other individuals engaged in UDMHI development, dissemination, and research.

#### Our Results in the Context of Existing Studies on UDMHI User Demographics

To put our results into context, we believe it is useful to compare our findings with those of other studies of publicly available and free UDMHIs targeting adults. In Table 4, we have included results from 6 other studies of real-world users of UDMHIs targeting depression or anxiety that reported age or gender information—these include the 5 studies referenced in the review paper by Fleming et al [3] as well as 1 subsequently published study [10]. Several similarities and contrasts between the users of the VA UDMHIs and users of other UDMHIs are worth noting. First, the percentage of VA UDMHI users who were men (3899/7068, 55.16%) was higher than that of any other UDMHI. This likely reflects the fact that most US veterans (ie, the target audience for the VA UDMHIs) are men and is consistent with the possibility that veterans were overrepresented among the VA UDMHIs’ users. Second, the median age of VA UDMHI users (35-44 y) was similar to that of users of most other UDMHIs with the exception of the users of *Hdep*, a web-based intervention for women in Mexico experiencing negative mood [21]. Third, the annual number of unique visitors to the VA UDMHIs was similar to that of 4 other UDMHIs (*Anxiety Online*, *Hdep*, *MoodGYM Mark 1*, and *MyCompass*). However, many more unique visitors were reported for *MoodGYM Mark 2* (which at the time of writing is one of the most successful web-based UDMHIs) and *Happify* (a commercial product that includes both free and paid features). Fourth, the location of users was reported for only one other UDMHI, *Hdep*. This study found that the vast majority of *Hdep* users (95%) were located in *Hdep*’s country of origin, Mexico. This was consistent with the observation that 93.63% (27,789/29,681) of the VA UDMHIs’ users were located in the VA UDMHIs’ country of origin, the United States. This is an interesting finding given that individuals from other countries that share a common language could theoretically easily use *Hdep* (ie, other Spanish-speaking countries) and the VA UDMHIs (ie, other English-speaking countries), but this sort of cross-border use was not found to be common in practice. Finally, none of the other published studies reported on the devices used by their UDMHIs’ users. Thus, we could not judge whether the predominance of desktop and laptop computer use (18,528/29,752, 62.27%) by VA UDMHI users was typical for these other web-based UDMHIs.

**Table 4:** Comparison of US Department of Veterans Affairs (VA) unguided digital mental health intervention (UDMHI) user characteristics with those reported for other free and publicly available UDMHIs.

UDMHI	Visitors or registrations/y	Woman users (%)	Age (years), mean	Age (years), median range	Users from country of origin (%)^a^	Dates observed
VA UDMHIs^b^	9769	45	—^c^	35-44	93	2021
Anxiety Online [20]	4032	69	37	—	—	2009 to 2012
CBT^d^ Psych [25]	96	—	35	—	—	2011 to 2014
Happify [23]	1,472,808	90	—	35-44	—	2014 to 2016
Hdep [21]	7020	84	—	18-30	95	2009 to 2013
MoodGYM Mark 1 [22]	5674^e^	60	36	34	—	April 1, 2001, to September 27, 2001
MoodGYM Mark 2 [19]	61,620	66	—	30-34	—	2006 to 2007
MyCompass [10]	8391	66	42	—	—	October 2011 to October 2012

^a^Country of origin refers to the country in which the UDMHI itself was developed.

^b^The numbers here reflect the weighted average across all 3 VA UDMHIs.

^c^This value could not be computed using the information available in the manuscript.

^d^CBT: cognitive behavioral therapy.

^e^This number only includes individuals who registered to use the website and does not include the college students who visited the website as part of a college psychology course assignment.

### Limitations

Our study has several important limitations. First, we had no way of knowing what proportion of the VA UDMHIs’ users were veterans. Thus, although it could be the case that differences between the demographics of the VA UDMHIs’ users and the US veteran population are due to veterans in certain demographic groups (eg, women and younger veterans) being more likely to use the UDMHIs than other veterans, the observed difference could also be because civilians, who are more likely than veterans to be woman and younger, frequently used the UDMHIs and, thus, inflated the proportion of woman and younger users. A second limitation of our study is that the Google Analytics data used in this study, like any web analytics data, inherently include some degree of measurement error. As noted earlier, the UDMHIs’ unique user counts could have been affected by extraneous factors such as users deleting browser cookies in between visits (which would artificially increase the unique user count) or multiple users using the same computer (which would artificially decrease the unique user count) [61].

Another inherent limitation of the precision of the Google Analytics data is that the demographic data are estimates based on the cookies present on the users’ computers. As we have previously noted, an existing study by Tschantz et al [60] found that such demographic estimates are fairly accurate when compared with self-reported demographic data; however, the possibility remains that the demographic data reported in our study might include inherent measurement error. One specific form of measurement error that might be of concern is whether individuals of certain genders or age groups might be more likely to engage in behaviors (eg, opting out of web analytics tracking) or use technologies (eg, AdBlock) that make it less likely that Google Analytics will produce a prediction for their gender or age. Thus, individuals in these gender or age groups would be underrepresented among the individuals with gender and age estimates and overrepresented among those without gender and age estimates. Reassuringly, an analysis by the authors of this paper using data reported in the study by Tschantz et al [60] found that age and gender were not significantly associated with the likelihood of being logged into a Google account at the time of participating in the study, which was the most important factor in determining whether Google Analytics provided an estimate of an individual’s gender and age category (see Tables S4 and S5 in Multimedia Appendix 1 for details of this analysis). However, other research has found that demographic variables are associated with the likelihood of using privacy-enhancing technologies (eg, a study found that men were more likely to use an advertisement blocker [64]), so it remains possible that certain gender or age categories were underrepresented in the 24.12% (7068/29,306) of individuals with estimated demographics in our sample.

Reassuringly, however, the VA UDMHIs’ user gender and age distributions were consistent with the demographic characteristics of the mental health problems that each UDMHI purported to help with. This congruence between the demographics of users and the expected demographics of the targeted mental health problem is a pattern we would expect if the demographic estimates accurately described the individuals visiting these websites. For example, men in the youngest age category (18-24 y) were overrepresented among AIMS users compared with users of the other 2 UDMHIs, which is consistent with the demographics of problematic anger in the general population [65]. Similarly, PTBS users were more likely to belong to the oldest age category (ie, ≥65 y), which, again, is consistent with the demographics of sleep disorders in the general population [66]. This observed consistency between the known demographic correlates of disorders and the estimated demographics of the users of the VA UDMHIs targeting the symptoms of those disorders provides circumstantial support for the validity of these Google Analytics–derived gender and age estimates.

The final limitation of our study that we wish to mention is that we focused our analysis on the demographics of users of the VA UDMHIs and did not analyze data on the behavior of users visiting the VA UDMHIs (eg, average time spent on web pages and average number of repeat visits). We limited our analyses to user demographic data as (1) we believe that user demographics are an important topic to study and that it made sense to limit the scope of this study to them and (2) although Google Analytics does allow a website developer to collect some user behavior data, the VA UDMHIs web pages were not designed in such a way as to ensure valid and readily analyzable data on user behaviors, thus raising concerns about the validity of analyses on user behavior variables. In contrast, within the limitations described in this paper, we believe that the demographic data collected using Google Analytics for these UDMHIs were valid and could provide a valid description of the demographics of their users.

### Recommendations for Other UDMHIs Targeting Similar Populations

Although the limitations of web analytics data must be kept in mind, we nevertheless believe that several of the findings reported in this paper are of relevance to the developers of other UDMHIs. First, our finding that both genders and all age groups were substantially represented among the users of the 3 VA UDMHIs suggests that developers of veteran-focused UDMHIs should not assume that all of their veteran users will be men aged >55 years. Our findings suggest that man and woman veterans of all ages are likely to use veteran-focused UDMHIs. Given the existing research finding that user engagement is increased when UDMHI users are able to identify with the characters, situations, and values portrayed in a UDMHI [1], UDMHI creators should endeavor to understand the characteristics and life experiences of their target audiences and represent those characteristics and life experiences in their interventions. Second, we found that most users accessed VA UDMHIs via desktop or laptop computers, which suggests that there is a population of individuals willing to access VA UDMHIs using such devices. We should note that, although the VA UDMHIs were built in such a way that their interface would adapt itself to the smaller screen of a mobile or tablet device if accessed from one, the VA UDMHIs did not have dedicated mobile apps available in mobile app stores. Thus, it is possible that a greater proportion of the VA UDMHI users would have used mobile and tablet devices to access the VA UDMHIs if dedicated apps were available to use. Nevertheless, we still believe that the large number of users who did access our VA UDMHIs using desktop or laptop computers suggests that it can be useful to design UDMHIs that are accessible via these devices.

### Conclusions

In this paper, we describe individuals who visited 3 free and publicly available web-based UDMHIs hosted by the VA in the year 2021. Using Google Analytics, we were able to gather information about the total number of users as well as their characteristics, including the devices they used to access the UDMHIs, their estimated age and gender, and their likely geographic location. On the basis of our findings, we suggest that individuals engaged in UDMHI development and research consider using web analytics software to collect user data for use in quality improvement projects and research. Given that many web analytics software packages include free-to-use features and require only minimal additional software coding to be integrated into an existing website, collecting such data requires a relatively small time and resource commitment. Given the relatively small amount of published data on the real-world use of publicly available UDMHIs, the collection and publication of such data will provide useful insights into the real-world use of UDMHIs. The broad adoption of Google Analytics and similar web analytics technologies to optimize websites to influence visitor behaviors (eg, increasing the likelihood that users purchase products, make donations, or read content) suggests that, despite their limitations, web analytics data provide useful and actionable information about users that could potentially be used to help increase user engagement with web-based UDMHIs.

However, an important issue to evaluate when considering the use of web analytics technologies are privacy concerns. Although the data gathered by Google Analytics may only include nonidentifying descriptions of users (eg, their age range, gender, and visits to a website), it remains possible that users will, for example, receive targeted advertisements related to mental health care following visits to mental health–related websites that use Google Analytics and similar software. More concerningly, several commercial mental health–related websites and apps have been found to package user-entered mental health–related data (eg, depression measure scores) along with web analytics data and sell it to advertising and data broker companies for use in targeted advertising [67]—such practices are now facing legal enforcement actions and legislative scrutiny [68]. UDMHI developers will need to stay abreast of the rapidly developing areas of UDMHI web analytics data law, privacy, and ethics and ensure that the web analytics tools they use comply with relevant institutional, legal, and ethical guidelines.

## Appendix

Multimedia Appendix 1 Population pyramids, accuracy calculations for age and gender, raw user counts for each unguided digital mental health intervention in each gender by age category, and association of gender and age with the likelihood of being logged into Google services.

## Abbreviations

AIMS: Anger and Irritability Management Skills
CBT: cognitive behavioral therapy
CBT-I: cognitive behavioral therapy for insomnia
MF: Moving Forward
PTBS: Path to Better Sleep
UDMHI: unguided digital mental health intervention
VA: US Department of Veterans Affairs
VTP: Veteran Training Portal

## Data Availability

US Department of Veterans Affairs policy prevents us from publicly releasing the data used in this study. If the reader has any concerns regarding the data or analyses conducted in this manuscript, they are invited to contact ATR to discuss gaining access to the underlying data and analyses.
